# Children’s Drawing of Plant Life in the Time of COVID-19: An Analysis of the Changes Related to Content and Colour over a Two-Year Period

**DOI:** 10.3390/children9060862

**Published:** 2022-06-10

**Authors:** Ilargi Zaballa, Maria Merino, José Domingo Villarroel

**Affiliations:** 1 Faculty of Education Bilbao, University of the Basque Country (UPV/EHU), 48940 Leioa, Spain; ilargi.zaballa@ehu.eus; 2 Faculty of Science and Technology, University of the Basque Country, (UPV/EHU), 48940 Leioa, Spain; maria.merino@ehu.eus

**Keywords:** early education, drawings, biological literacy, plant life, biology, learning loss

## Abstract

This study analyses the depictions that a sample of young children completed to express their knowledge of plant life at two different times, two years apart. The pictorial content is examined by the complexity of the depictions of flora as well as the range of colour that the children in the sample chose. The study presents the changes that occurred in the children’s illustrations of plants after 24 months. The conclusions are discussed in view of the data that preceding studies provide on the subject of botanical literacy in childhood, and raise the hypothesis that the unexpected results obtained in the study might reflect a learning loss in the understanding of the plant world as a consequence of the school closures that followed the outbreak of the COVID-19 pandemic.

## 1. Introduction

Today, children are born into a world that faces crucial environmental concerns, including climate change, pollution, and the loss of biodiversity. All over the world, scientists, social leaders and the media consider these topics as global concerns, but they are also local issues integrated into educational subjects in schools [1]. Thus, primary school education around the world has shown an increasing movement towards providing children with opportunities to understand current environmental challenges and how to act sustainably. To make this connection, early education school programs that promote knowledge and interest in the plant world appear to be a relevant objective for development [2,3]. Actually, if further interest in learning about plants does not arise by around the age of 9, children become limited in terms of botanical understanding [4]. This fact strongly suggests that early education is a critical time for children to learn about plants and, not surprisingly, botanical learning is an essential part of the natural sciences curriculum in primary schools [5].

Examining how knowledge, curiosity and thoughts about plants and their environments evolve in childhood is also an area of study that currently attracts much attention in the field of science education research [4]. With attention to this goal, recent research points out that the study of children’s drawings is a useful tool to examine children’s ideas on the concept of plants [6], as well as to identify faulty knowledge about botanical concepts [7]. A common assumption shared by studies involving the study of children’s depictions is the belief that children express their cognitive and emotional processes in their drawings. For that reason, a careful analysis of the pictures that they create provides researchers with information related to, among other aspects, children’s thinking and conceptual development [8].

In this regard, recent research indicates that children’s representations of plant life relate to their comprehension of the fact that plants are living things [9], as well as to their conceptions about the structure and function of plants [6]. In addition, the colours that children choose in their drawings appear related to their level of understanding of plant life in such a way that the colour palette they choose to use in their drawings of flora becomes narrower and more realistic as their understanding improves [10]. It has also been stated that young children tend to depict animals more often in close connection with plants, for instance, feeding on flowers or living in green habitats, as their drawings display a deeper knowledge of the plant world. This observation is coherent with the belief that the process of grasping the ecological connections between plants and animals arises before the age of eight [11]. Furthermore, one study that followed young children’s representations of the plant world over a one-year period accounted for three major changes in the drawings: first, children tended to draw a wider range of plant specimens after 12 months; second, they depicted some anatomic parts of plants in more detail; and finally, they included abiotic factors, such as the sun, rainfall or soil, in their pictures more often [12].

It is worth noting that no other study has addressed the evaluation method of how the drawings that children produce, with the aim of expressing their knowledge of the plant world, change over time, and that no research project has analysed how children’s drawings change over a period of more than 12 months. Consequently, this study starts from the idea that analysing the early childhood development of graphical expressivity regarding plant life more closely is necessary to obtain a better understanding of how botanical literacy evolves in early childhood. Thus, the research question that this research project posits is as follows: What are the main changes that children under ten depict in drawings when trying to express their understanding of plants over a 2-year time period?

To that end, this study examines the changes related to pictorial content and the utilisation of colour over a two-year time period in the drawings in which young children display their knowledge of plant life.

Specifically, the objectives that the study intends to achieve are as follows:

1. To identify and classify the pictorial elements that the children in the sample depict to express their understanding of the plant world in accordance with the categories indicated by previous research;
2. To identify the colours that the children in the sample choose in their representations of the plant world and measure the area covered with each of the colours;
3. To compare the drawings that the children completed in 2019 to express their knowledge of plant life with those that the same sample of children drew two years later. This comparison will involve both the pictorial content of the drawings and the analysis of the colours that the children chose to paint their representations.

On a separate but not unrelated matter, it is worth mentioning the exceptionality of the time window during which the present study was completed. As will be explained in more detail in the methodology section of this paper, the COVID-19 pandemic shook Spanish society and the entire world between the two moments in which the drawing activities involved in this study were carried out. Accordingly, although this exceptional circumstance was not anticipated when this study was defined, the loss of schooling that the children suffered due to pandemic lockdowns will be an unavoidable issue with the data collated in the present study throughout this paper.

## 2. Materials and Methods

### 2.1. Sample

The sample included 140 drawings that 70 children completed at two different times. The first drawing activity was carried out in June 2019, and the children carried out the second activity 2 years later in June 2021.

All the children involved in the study attended a state-run school located in the municipality of Getxo (the Basque Country, Spain). This educational centre is a medium-sized primary school with approximately 250 children, located on the outskirts of the urban area and bordering a rural area. The school was chosen by the criterion of accessibility to the research team.

When the 70 children completed the first drawing activity in 2019, 32.9% were in their penultimate course in preschool education, 32.9% were in their last course in preschool education and 34.3% were in their first academic year in primary education. By 2021, all children had moved up two educational levels with the same class group.

All the children involved in the first pictorial activity in 2019 also completed the second drawing two years later.

### 2.2. Data Collection

This study analyses the two drawings that each child involved in the sample completed during two individual interviews, two years apart. A single researcher oversaw the individual interviews with the children, and this academic was the same person in both interviews.

The process for conducting the interviews matched the methodology proposed by prior research in the study of young children’s depictions of flora [9,13,14,15]. This process involves two different stages. Initially, the researcher, with the aid of the teacher of the classroom, presents the activity in a meeting with all the children. The researcher tells the children a short tale about a character who is eager to know more about plants, flora and vegetables and asks the children to help her to grasp what plants are like, what they need to live and grow and where they exist. The researcher accompanies the story with a puppet that she has taken to the meeting with children. The children are asked to explain everything that they know about plants by means of a drawing that they will complete in the next few days.

Subsequently, individual interviews with each of the children in the sample are carried out. These individual meetings take place in a small room attached to the classroom. At the beginning of the meeting, the researcher helps the child to remember the objective of the pictorial activity by showing them the puppet and reminding them of the wish that the character in the tale had to know more about plants. Then, the researcher gives the child a paper and some pens so that she can begin the drawing. During the drawing activity, no information is given to the child regarding the content that could be drawn. After the child expresses that the drawing is finished, the researcher offers him or her 12 different felt-tip pens arranged randomly. The colours of the felt-tip pens are as follows: black, brown, purple, grey, light blue, dark blue, red, yellow, orange, light green, dark green and pink. The interview is completed when the child in the meeting indicates that the drawing with colouring is finished. Meetings usually took between 10 and 15 min, and the children were generally excited to participate in the activity.

### 2.3. Target Study Variables

**Figure A1:**
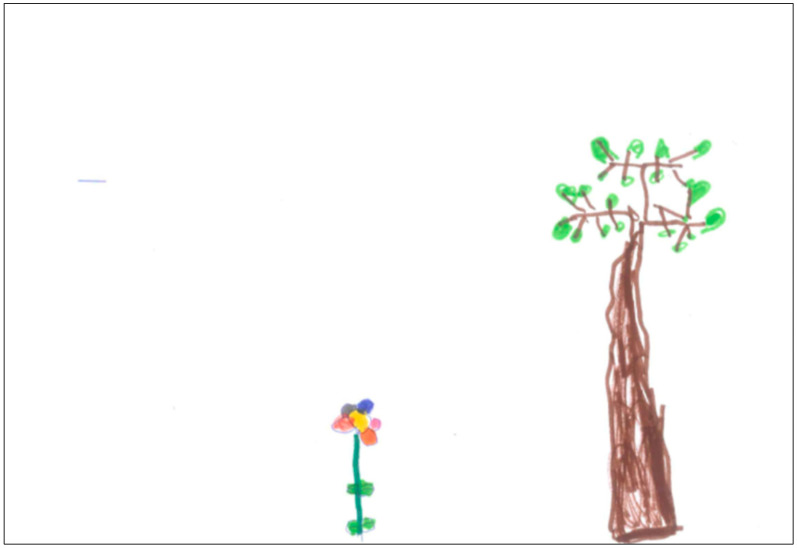
The drawings on the topic of plant that a 5 year old child completed in 2019.

**Figure A2:**
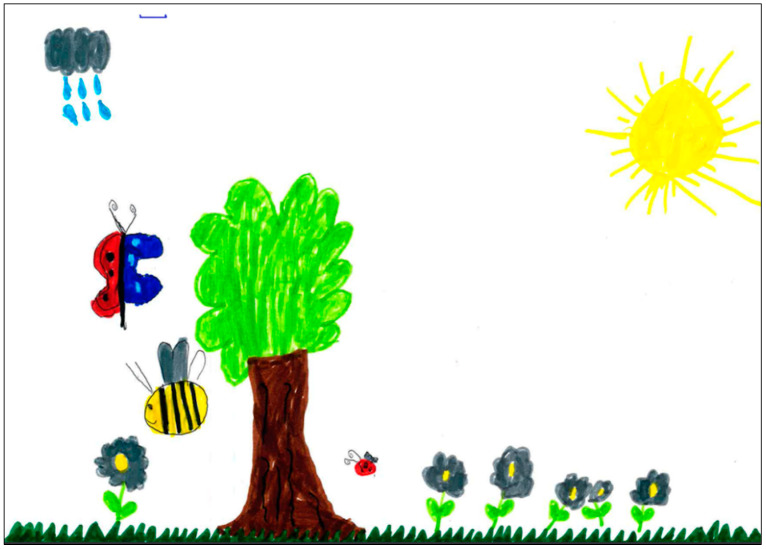
The drawing on the topic of plant that the same child indicated in Figure A1, completed in 2021.

**Figure A3:**
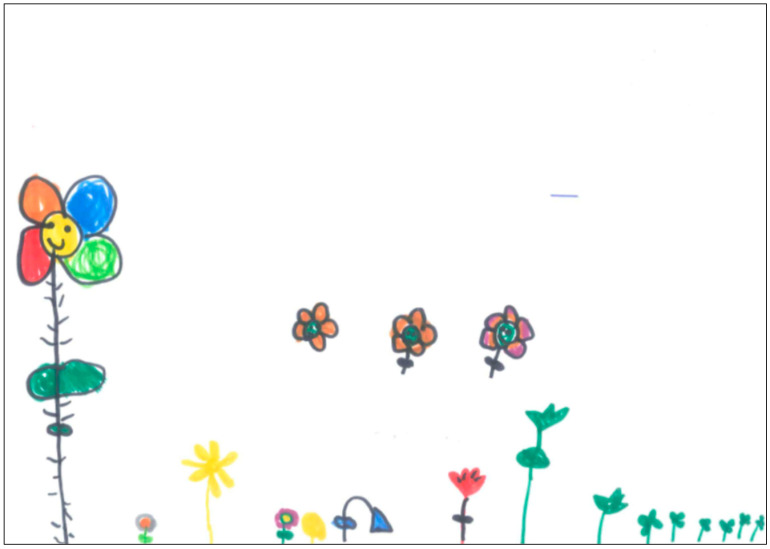
The drawings on the topic of plant that a 6 year old child completed in 2019.

**Figure A4:**
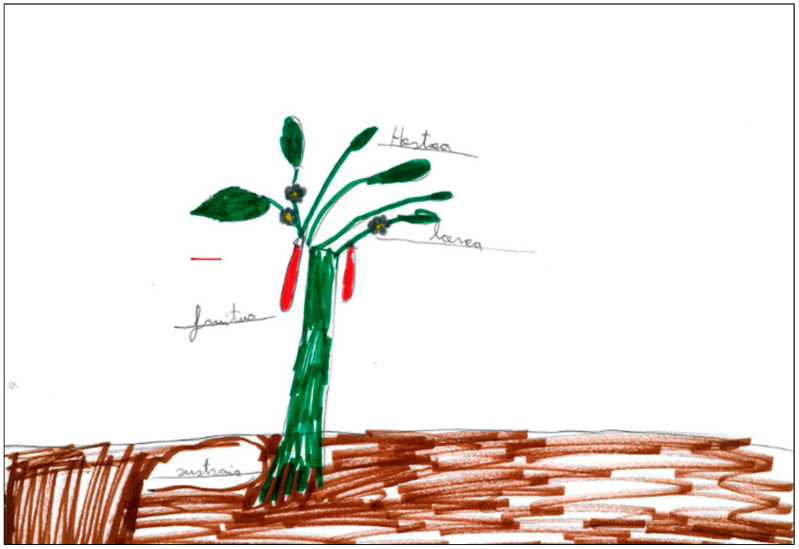
The drawing on the topic of plant that the same child indicated in Figure A3, completed in 2021.

This research project quantitatively examines the pictorial content and the utilisation of different colours in the drawings that the children involved in the study carried out to express their understanding of plant life at two different moments, 24 months apart. Four examples of the drawings analysed are displayed in Appendix A of the paper (see Figure A1, Figure A2, Figure A3 and Figure A4).

The independent variable that this research project considers is the year that the drawings were completed, that is, in 2019 or in 2021.

Moreover, the study identifies two dependent variables. The first one is the utilization of each of the 12 colours and the total area coloured. The second dependent variable is the number of different pictorial elements within the five categories described in Table 1. These five categories have been proposed by previous research to examine the drawings that young children depict on the issue of plant life [16,17].

### 2.4. Statistical Procedures

The Wilcoxon signed-rank test was used to analyse the differences between the depictions that the children in the sample created at the two different drawing activities (in 2019 and in 2021) using the pre-assigned pictorial categories. The correlation coefficient r was considered to measure the effect size [18,19] and the cut-off level indicating a low correlation between the variables was set to 24 [20].

The level of significance observed for refusing the null hypotheses was 5%. The statistical methods were performed by SPSS software, version 22, and the software R version 3.5.0 was employed to design the figures in the paper [21].

### 2.5. Ethics

The Ethics Committee for Research on Human Beings at the University of the Basque Country approved and tracked the present research project (M10_2016_247MR1_VILLARROEL VILLAMOR).

The board of directors of the school as well as the teachers were informed in advance about the objectives and methodology of the research project, and they approved the participation of the educational centre in the study. Moreover, the research group informed families of the children involved in the study regarding the goals and procedure of the study. The families had to give written permission to allow their children to participate in the research project and they could opt their child out of participating in the study.

No pictures or recordings were taken during the interviews with the children in the sample.

## 3. Results

The results section is divided into two parts. Initially, the results linked to the whole sample are introduced, and subsequently, the outcomes are spelled out in accordance with the gender variable. In both cases, information is provided regarding, first, the study of the pictorial content and, second, the analysis of the surface coloured in the drawings.

### 3.1. Regarding the Examination of Pictorial Content

In total, 548 pictorial elements were identified in the 140 depictions under study; 42.7% of them were found in the 70 illustrations included in the first drawing activity (in 2019), and 57.3% were found in the activity that the children completed two years later. Table 2 shows an overview of the relative frequencies related to the five categories of pictorial elements in the whole sample.

Moreover, Table 3 introduces the descriptive statistics of the frequency of the pictorial categories in drawings completed in 2019 and 2021. The abiotic factor category (Wilcoxon *t* test = −2.92; *p* value < 0.05; r = 0.25) is the only one that displayed significant differences when comparing the pictorial content in the drawings completed in 2019 with those realised two years later.

Finally, it should be noted that there were no significant differences between each age group relating to the pictorial content of the drawings made in 2019 and those carried out 2 years later.

### 3.2. Regarding the Examination of the Coloured Area

In total, the 70 children in the sample covered 15,404 cm^2^ with colour in the two pictorial activities that they completed in 2019 and 2021. Table 4 introduces the total area coloured with each colour in reference to all 140 drawings included in the sample.

**Figure 1:**
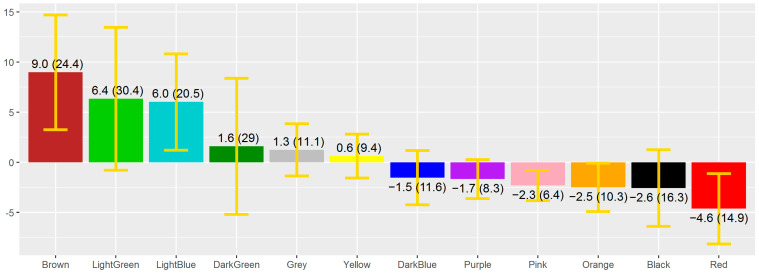
Mean difference between the coloured area (standard deviation) for each of the 12 colours available to children in the pictorial activities of 2021 and 2019 (cm^2^) and confidence interval for the mean difference (95%).

Moreover, on average, the 70 children in the sample painted a surface of 59.2 cm^2^ (SD = 60.2 cm^2^) in the drawings completed in 2019 and 160.9 cm^2^ (SD = 67.3 cm^2^) in those depicted two years later, in 2021. Figure 1 illustrates the mean difference between the years 2021 and 2019 regarding the area coloured with each colour (i.e., the mean area coloured in 2021 minus the mean surface coloured two years earlier). Along with this, the standard deviation and confidence interval for the mean difference (95%) are also displayed.

The study of the comparison of the coloured surfaces in the two drawing activities showed significant differences in relation to the red (Wilcoxon *t* test = −2.94; *p* value < 0.01; r = 0.25) and pink (Wilcoxon *t* test = −3.44; *p* value < 0.01; r = 0.29) colours.

## 4. Discussion and Conclusions

The examination of the content of the drawings in the sample (*n* = 140) indicated that a high proportion of the pictorial elements identified were in line with the theme of the plant world (more than nine out of ten). Thus, the representation of specimens of plants and their anatomical structures accounted for more than half of the pictorial elements that the children drew. Along with this, almost three pictorial elements in ten illustrated abiotic factors that play a crucial role in plant life (such as soil, the sun, or rainfall), and in addition, nearly 10 percent of the elements depicted represented other living things.

These findings suggest that the children in the sample did not draw casual or accidental representations. In contrast, the evidence presented is consistent with the idea that, before translating their thoughts into drawings, young children intentionally selected concepts and perceptions belonging to the botanical domain from their body of knowledge. This assumption is also supported by previous research [6,16,22,23], and is consistent with the belief that, before the age of 9, children are already engaged in a process of grasping biological phenomena and plant life.

The preceding considerations also seem to apply to the range of colours chosen by the children in the sample. Thus, blue, green, brown and yellow have been pointed out by previous researchers as the most frequent colours in drawings of the plant world in early childhood because these are the colours that children use to illustrate the sky, vegetables and plants, tree trunks, and the sun, respectively [10]. It is worth noting that these same colours are, precisely, those most commonly used in the sample. Additionally, orange, pink and purple were colours that children often selected to give colour to pictorial motifs that covered small surfaces in their drawings, for example, fruits or flowers, and these colours were among those that displayed a smaller total area in the sample analysed.

Another specific point concerns the significant differences in the content associated with one single pictorial category (abiotic factors) between the two drawing activities. Accordingly, the children involved in this study were more likely to represent, for instance, the sun, soil, rainfall or waterways in the pictures produced in 2021 than in those drawn two years earlier.

Previous research, however, indicates that after a 12-month time period, the representations of plant life that young children create display significant changes associated with the samples of plants and the external structures of plants, as well as abiotic factors [12]. This observation is provided by studies indicating that young children’s pictorial representations of the plant world tend to evolve to include a greater variety of specimens of flora, with more details of the anatomical structure of plants as well as more abiotic factors and depictions of animals in ecological connection with the plant world [24,25].

Overall, the evidence that this study introduces does not seem to be consistent with the data presented by previous research. This research project indicates that the change in the graphical expression of botanical understanding in early childhood seems to be limited to a single pictorial category related to the comprehension of the role that abiotic factors play in plant life. However, the evidence provided by previous research points out, first, that as they age, young children introduce into their drawings a more widespread change that involves pictorial categories such as samples of plants, external structures of plants, abiotic factors, and other living things and, second, that this change occurs in a noticeably shorter period of time.

The question arises as to why the drawings involved in the present study did not indicate that the children had achieved any conceptual advance regarding the comprehension of crucial components of botanical knowledge such as the diversity and variety of plant life, the anatomical details of plants, and the ecological connections between animals and plants. The fact that previous research shows that such conceptual achievements are accomplished by young children in a shorter period of time makes the above question even more relevant.

One cannot fail to consider that, although the children in the present sample carried out their first drawing activity before the beginning of the COVID-19 pandemic in March 2020, they completed their second drawing after schools were forced to take exceptional measures to protect public health. In this way, the second pictorial activity was undertaken after three months of school closures (March to June 2020), the summer break of 2020, and six months of progressive school opening with temporary closures of classrooms and educational centres running in morning shifts (September 2020 to March 2021).

There is currently a significant body of evidence that allows us to affirm that school closures due to the COVID-19 pandemic had a significant impact on students’ learning processes. Thus, a meta-analysis of papers that address the issue of the impact of the pandemic on academic performance concludes, first, that school closures due to the COVID-19 pandemic had an important negative average effect on the academic achievement of students in compulsory education and, second, that the younger the students, the more negative this effect [26]. Similarly, a study intended to measure the impact that the COVID-19 pandemic had on primary and secondary education in the Basque Country (the Spanish region in which the present study took place) accounts for significant learning losses as well as negative effects on students’ well-being [27].

In this context, it does not seem unreasonable to postulate that the closure of schools due to the COVID-19 pandemic could have had a negative impact on the development of the understanding of biological phenomena. Therefore, it is possible that children’s understanding of the plant world may not have developed as might have been expected under normal school conditions.

It is worth noting that the analysis of drawings is distinguished as a particularly effective method for demonstrating the development of the comprehension of biological phenomena and, particularly, plant life in early childhood [17,25]. Consequently, the data provided by this study are consistent with the hypothesis that the drawings in the sample reflect a learning loss due to the school closures following the outbreak of the COVID-19 pandemic. In this regard, it should be noted that, during the lockdowns, environmental and outdoor school activities were moved to the virtual world, and although online activities played a key role in maintaining access to school, the truth is that this situation implied a loss of sensory experiences and emotional connection with nature [28]. Thus, crucial areas of the comprehension of flora, such as the variety of plant specimens, the anatomical details of plants, and the ecological links between plants and animals, could have been affected by the lack of schooling in the 2019/20 academic year.

## 5. Limits

The assumption indicated in the conclusions chapter is certainly limited in the con-text of the present study. First, this research project cannot rule out the effect that other factors not accounted for in this study could have had in shaping the particular characteristics of the drawings that the children depicted. In addition, this study does not specify any cause–effect relationship between the pictorial characteristics of the drawings analysed and the fact that the children missed several months of schooling. Thus, this paper accounts for an overlap in time between school closures and the fact that the evolution of the knowledge regarding the world of plants expressed by children differed importantly from what was expected.

Contrasting the information presented in this paper with the data provided in forthcoming studies regarding learning losses due to the COVID-19 pandemic will presumably confirm the line of thought presented in this research project.

Finally, one cannot fail to consider the need to develop teaching proposals intended to promote botanical literacy in early childhood in response to the learning losses to which this article refers. In this sense, it is worth mentioning the relevant role that storybook-based educational activities play in initiating young children in the understanding of formal science [29,30].

## Figures and Tables

**Table 1:** A description of the pictorial categories considered in the study, as well as some examples of images found in the sample.

Categories	Theme	Pictorial Elements
Samples of plants	Variability and diversity of the plant world	Flowers, vegetables, grass and trees
External structure of plants	Description of anatomical parts of the specimens of plants	Fruits, leaves, roots and seeds
Abiotic factors	Inanimate entities in the environment that affect plant life	Clouds, mountains, rivers, rainwater, the sun, soil
Other living things	Living components of the environment	Wild vertebrates: dogs, rabbits, people, bears Invertebrates: bees, wasps, butterflies, ladybugs Other nonanimal taxa
Unrelated to the plant world	Objects without or with a tenuous link to plant life	Toys, geometric forms, vehicles, stars, buildings, and hearts

**Table 2:** Relative frequencies (%) of pictorial elements broken down by pictorial categories in the whole sample (*n* = 140).

Categories	%
Sample of plants	47.8
Abiotic factors	29.6
External structure of plants	4.8
Other living things	8.6
Items unrelated to plants	8.9

**Table 3:** Statistical descriptors of the frequency of the pictorial categories under study in the first drawing activity (2019), as well as two years later.

**Categories**	2019 (*n* = 70)				2021 (*n* = 70)			
	Median	Min-Max	$\bar{X}$	SD	Median	Min-Max	$\bar{X}$	SD
Sample of plants	2.0	0–9	2.0	1.5	2.0	0–5	2.1	1.1
Abiotic factors	0.0	0–5	0.8	1.1	1.0	0–6	1.3	1.5
External structure of plants	0.0	0–2	0.1	0.3	0.0	0–3	0.2	0.6
Other living things	0.0	0–4	0.2	0.6	0.0	0–4	0.4	0.9
Items unrelated to plants	0.0	0–6	0.5	1.0	0.0	0–3	0.4	0.8

**Table 4:** Total surface colour (cm^2^) with each of the twelve different felt-tip pens that the children had at their disposal in the two pictorial activities (*n* = 140).

Light Blue	Light Green	Brown	Dark Green	Yellow	Red	Black	Orange	Dark Blue	Grey	Pink	Purple
7191	2078	1609	1390	786	540	416	356	336	322	244	223

## Data Availability

Data available on request from the authors.

## References

[1] Hedefalk M., Almqvist J., Östman L. (2015). Education for sustainable development in early childhood education: A review of the research literature. Environ. Educ. Res..

[2] Link-Pérez M.A., Schussler E. (2013). Elementary botany: How teachers in one school district teach about plants. Plant Sci. Bull..

[3] Stagg B.C., Verde M.F. (2019). Story of a Seed: Educational theatre improves students’ comprehension of plant reproduction and attitudes to plants in primary science education. Res. Sci. Technol. Educ..

[4] Beasley K., Lee-Hammond L., Hesterman S. (2021). A Framework for Supporting the Development of Botanical Literacies in Early Childhood Education. Int. J. Early Child..

[5] Chien Y., Su Y., Wu T., Huang Y. (2019). Enhancing students’ botanical learning by using augmented reality. Univers. Access Inf. Soc..

[6] Anderson J.L., Ellis J.P., Jones A.M. (2014). Understanding early elementary children’s conceptual knowledge of plant structure and function through drawings. CBE—Life Sci. Educ..

[7] Bartoszeck A.B., Cosmo C.R., Dasilva B.R., Tunnicliffe S.D. (2015). Concepts of plants held by young Brazilian children: An exploratory study. Eur. J. Educ. Res..

[8] Cachón-Zagalaz J., Sanabrias-Moreno D., Sánchez-Zafra M., Lara-Sánchez A.J., Zagalaz-Sánchez M.L. (2021). The Physical Education Class Perceived by Schoolchildren from 6 to 8 Years Old Expressed through Drawings. Children.

[9] Villarroel J.D., Infante G. (2014). Early understanding of the concept of living things: An examination of young children’s drawings of plant life. J. Biol. Educ..

[10] Villarroel J.D. (2016). Young Children’s Drawings of Plant Life: A Study Concerning the Use of Colours and its Relationship with Age. J. Biol. Educ..

[11] Villarroel J.D., Antón A., Zuazagoitia D., Nuño T. (2018). A Study on the Spontaneous Representation of Animals in Young Children’s Drawings of Plant Life. Sustainability.

[12] Villanueva X., Villarroel J.D., Antón A. (2021). Young children’s drawings of plant world: A cohort study analysing pictorial content. J. Biol. Educ..

[13] Villarroel J.D., Merino M., Antón Á. (2019). Symmetrical Motifs in Young Children’s Drawings: A Study on Their Representations of Plant Life. Symmetry.

[14] Villarroel J.D., Merino M., Antón A. (2021). Young children’s spontaneous use of isometric and non-isometric symmetries: A study regarding their unprompted depictions of plant life. J. Biol. Educ..

[15] Villarroel J.D., Sanz Ortega O. (2017). A study regarding the spontaneous use of geometric shapes in young children’s drawings. Educ. Stud. Math..

[16] Ahi B. (2017). The world of plants in children’s drawings: Color preferences and the effect of age and gender on these preferences. J. Balt. Sci. Educ..

[17] Zaballa I., Merino M., Villarroel J. (2021). Children’s Pictorial Expression of Plant Life and Its Connection with School-Based Greenness. Sustainability.

[18] Cohen J. (1992). Statistical power analysis. Curr. Dir. Psychol. Sci..

[19] Fritz C.O., Morris P.E., Richler J.J. (2012). Effect size estimates: Current use, calculations, and interpretation. J. Exp. Psychol. Gen..

[20] Lovakov A., Agadullina E.R. (2021). Empirically derived guidelines for effect size interpretation in social psychology. Eur. J. Soc. Psychol..

[21] R Core Team (2018). R: A Language and Environment for Statistical Computing.

[22] Atasoy V., Ahi B., Balci S. (2020). What do primary school students’ drawings tell us about their mental models on marine environments?. Int. J. Sci. Educ..

[23] Sanz Ortega O. (2015). Acercamiento a la comprensión del concepto de ser vivo en educación infantil. Ikastorratza e-Rev. De Didáctica.

[24] Villarroel J.D., Nuño T., Antón A., Zuazagoitia D. (2016). Un estudio en torno a comprensión infantil del mundo vegetal a través de sus dibujos [A study regarding young children’s understanding of the plant world through their drawings]. ENSAYOS Rev. De La Fac. De Educ. De Albacete.

[25] Villarroel J.D., Antón A., Zuazagoitia D., Nuño T. (2018). Young children’s understanding of plant life: A study exploring rural–urban differences in their drawings. J. Biol. Educ..

[26] König C., Frey A. (2022). The Impact of COVID-19-Related School Closures on Student Achievement—A Meta-Analysis. Educ. Meas. Issues Pract..

[27] Arenas A., Gortazar L. (2022). Learning loss one year after school closures: Evidence from the Basque Country. IEB Work. Pap..

[28] Rios C., Neilson A.L., Menezes I. (2021). COVID-19 and the desire of children to return to nature: Emotions in the face of environmental and intergenerational injustices. J. Environ. Educ..

[29] Kelemen D. (2019). The magic of mechanism: Explanation-based instruction on counterintuitive concepts in early childhood. Perspect. Psychol. Sci..

[30] Kalogiannakis M., Nirgianaki G., Papadakis S. (2018). Teaching magnetism to preschool children: The effectiveness of picture story reading. Early Child. Educ. J..

